# Supplementary figures and images for: Clostridium butyricum and Clostridium tyrobutyricum: angel or devil for necrotizing enterocolitis?
Source: mSystems. 2023 Nov 3;8(6):e00732-23. doi: 10.1128/msystems.00732-23 (PMC10734425; doi:10.1128/msystems.00732-23)

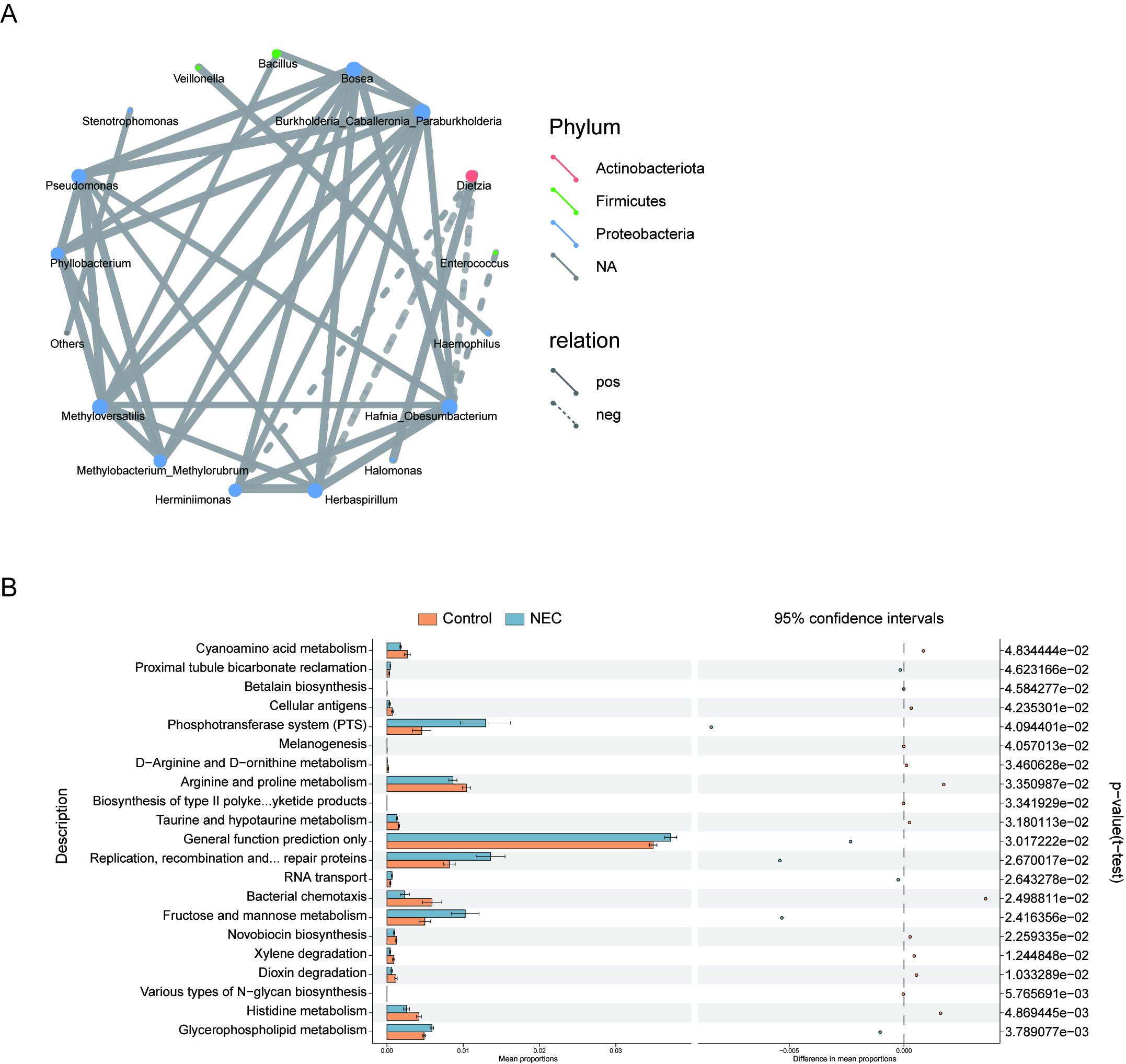

Supplement: Fig. S1 — Analysis of microbial networks. [file msystems.00732-23-s0001.tif]

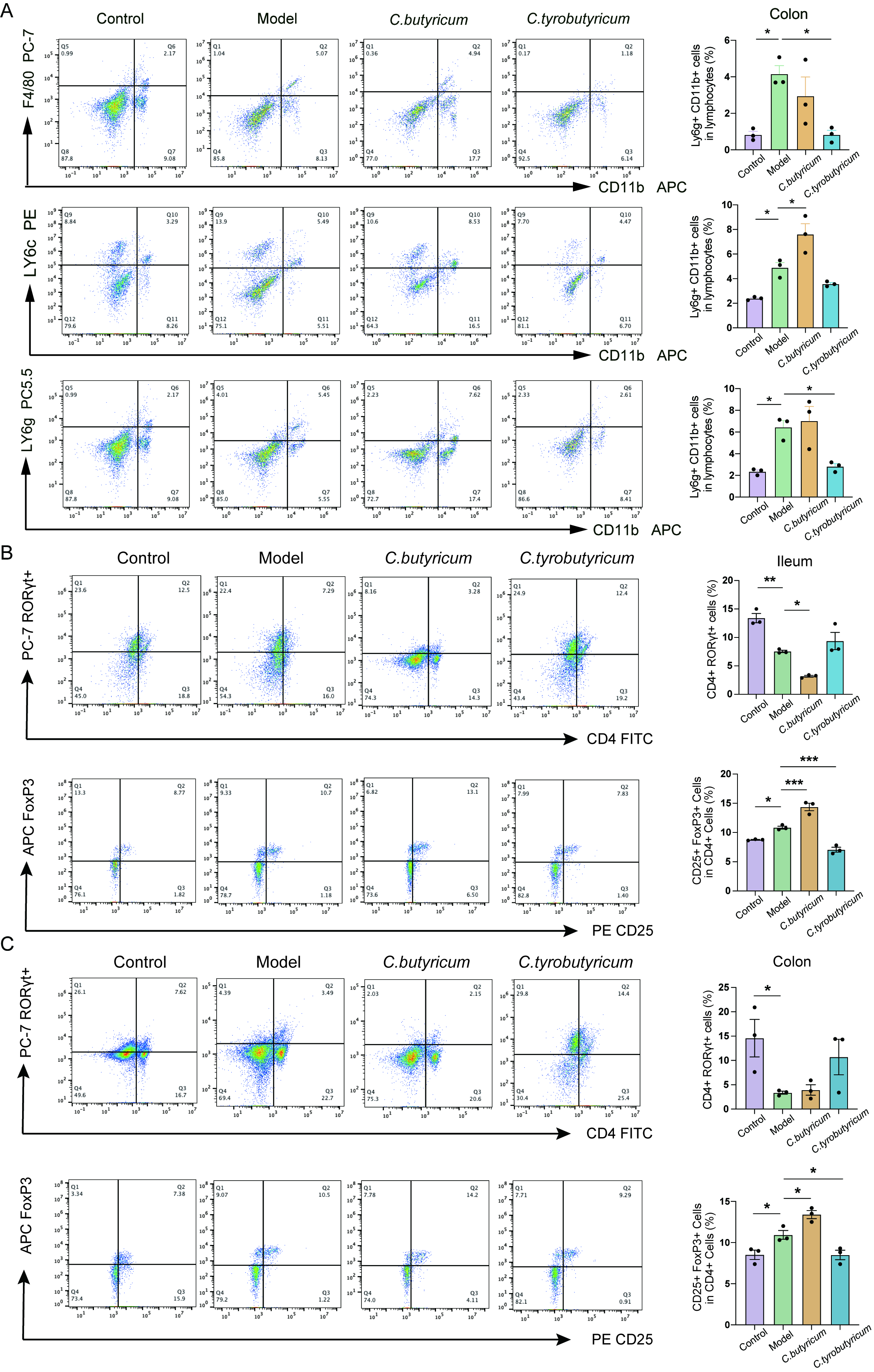

Supplement: Fig. S2 — Alleviation or aggravation of intestinal inflammation. [file msystems.00732-23-s0002.tif]

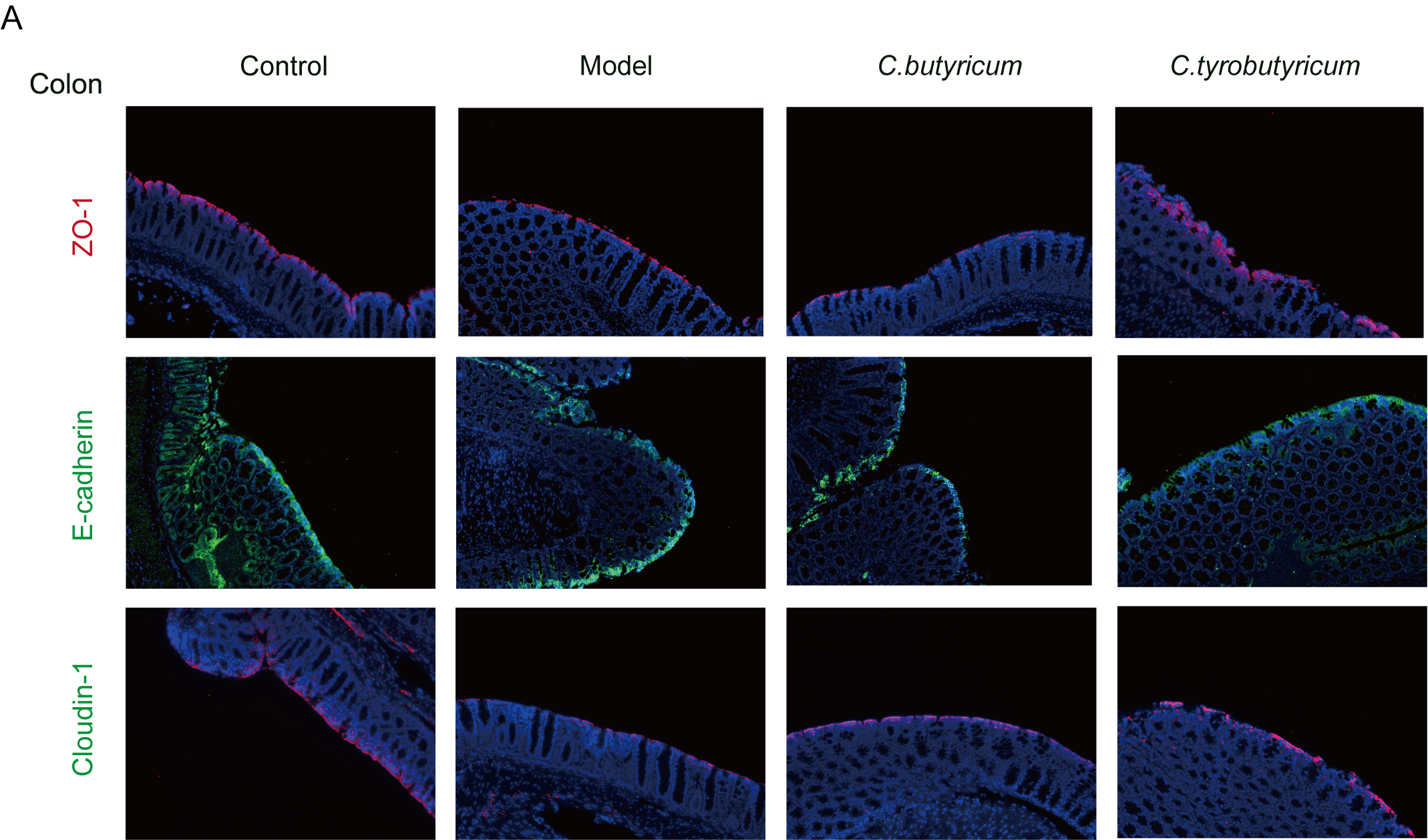

Supplement: Fig. S3 — Protection or disruption of intestinal barrier integrity. [file msystems.00732-23-s0003.tif]

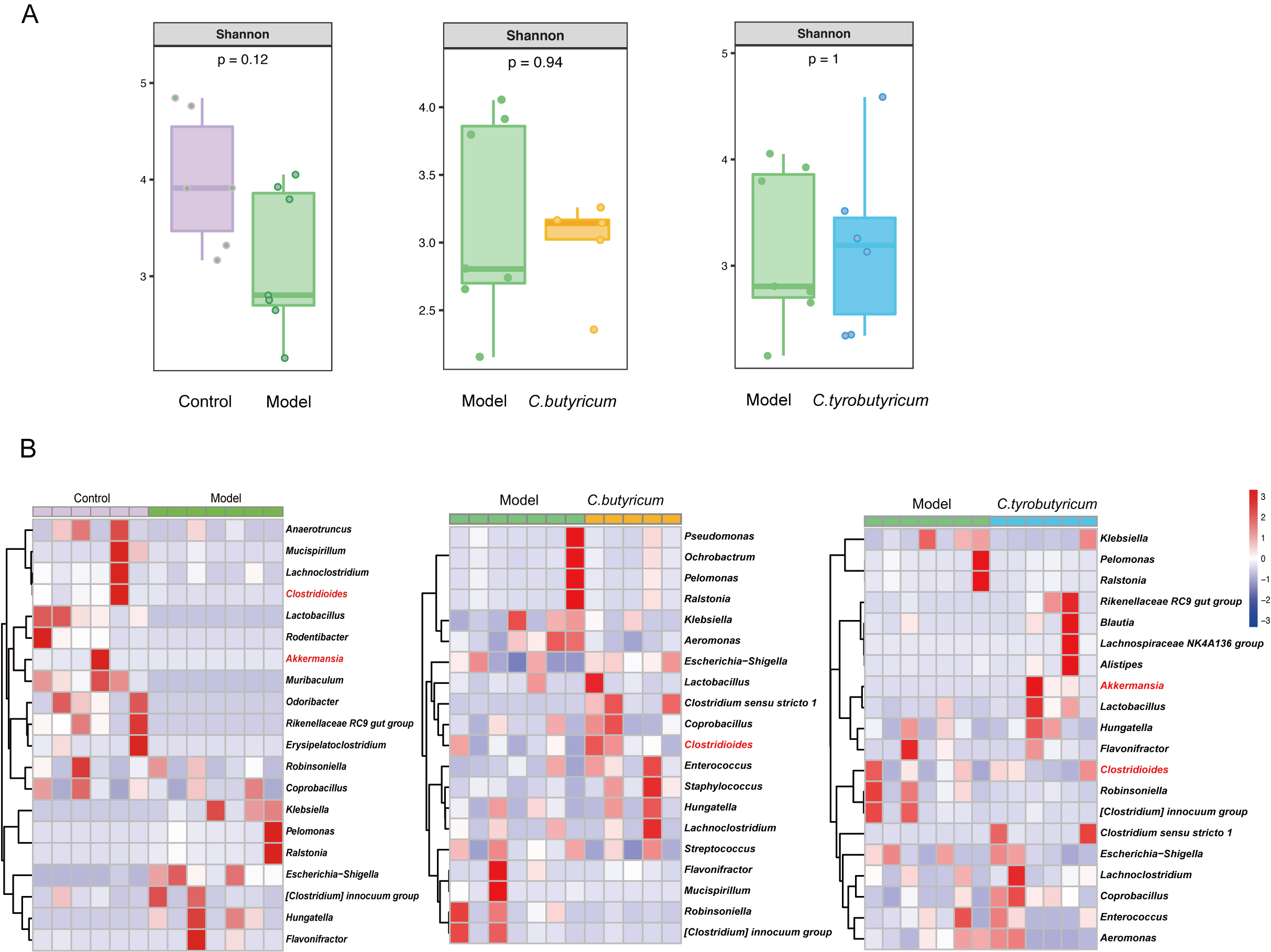

Supplement: Fig. S4 — Effects on NEC. [file msystems.00732-23-s0004.tif]
