# Supplementary material for: Clostridium butyricum and Clostridium tyrobutyricum: angel or devil for necrotizing enterocolitis?
Source: mSystems. 2023 Nov 3;8(6):e00732-23. doi: 10.1128/msystems.00732-23 (PMC10734425; doi:10.1128/msystems.00732-23)
Supplement: Supplemental legends — Legends to Table S1 and Fig. S1 to S4. [file msystems.00732-23-s0005.docx]

**Supplemental legends**

**Table. S1** Information of human samples related to materials and methods.

**Figure. S1** (A) The analysis of microbial networks present in the microbiome of infants' feces. (B) The PICRUSt2 predicted results shed light on the functional differences of the distinct microbiomes present in each group.

**Figure. S2** Intestinal inflammation was alleviated by *C.tyrobutyricum* but aggravated by *C.butyricum*. (A) Flow cytometric analysis of CD11b^+^F4/80^+^(Macrophages), CD11b^+^Ly6C^+^ (Monocytes) and CD11b^+^Ly6G^+^ (Neutrophils) cells in the colon tissues of mice (n=4). (B) Flow cytometric analysis of CD4+ RORγt+ (Th17) cells and CD25+ FoxP3+ (Treg) cells in ileum tissues (n=3) (C) Flow cytometric analysis of CD4+ ROR**γ**t+ (Th17) cells and CD25+ FoxP3+ (Treg) cells in colon tissues (n=3). Quantified results were shown as mean ± SEM. p-values were generated by one-way ANOVA with multiple comparisons. *p＜0.05, **p＜0.01,*** p＜0.001.

**Figure. S3** Intestinal barrier integrity was protected by *C.tyrobutyricum* but disrupted by *C.butyricum*. (A) Immunofluorescence analysis on ZO-1, E-Cadherin, and Claudin-1 in colon sections from different groups. Representative images were shown (100x). Scale bar: 100 µm.

**Figure. S4** The positive effect of *C.tyrobutyricum* and the negative effect of *C.butyricum* on NEC were associated with modulating the level of *A. muciniphila*. (A) Representative images of Shannon index of Control, model, *C.butyricum* and *C.tyrobutyricum* mice. (B) Heatmaps of bacteria of different groups at genus level. Each column corresponds to one sample.
